# Performance Evaluation of a Dense MEMS-Based Seismic Sensor Array Deployed in the Sichuan-Yunnan Border Region for Earthquake Early Warning

**DOI:** 10.3390/mi10110735

**Published:** 2019-10-29

**Authors:** Chaoyong Peng, Peng Jiang, Quansheng Chen, Qiang Ma, Jiansi Yang

**Affiliations:** 1 Institute of Geophysics, China Earthquake Administration, Beijing 100081, China; yangjiansi@vip.sina.com; 2 Sichuan Earthquake Administration, Chengdu 610041, China; 706650561@e-mail.com; 3 Beijing Gangzhen Instrument & Equipment Co., LTD., Beijing 102628, China; chenqs@geodevice.cn; 4 Institute of Engineering Mechanics, China Earthquake Administration, Harbin 150080, China; maqiang@iem.ac.cn

**Keywords:** earthquake early warning, shake map, MEMS, low-cost seismic sensor

## Abstract

With the last decades of development, earthquake early warning (EEW) has proven to be one of the potential means for disaster mitigation. Usually, the density of the EEW network determines the performance of the EEW system. For reducing the cost of sensors and building a dense EEW network, an upgraded low-cost Micro Electro Mechanical System (MEMS)-based sensor named GL-P2B was developed in this research. This device uses a new high-performance CPU board and is built on a custom-tailored Linux 3.6.9 operating system integrating with seismological processing. Approximately 170 GL-P2Bs were installed and tested in the Sichuan-Yunnan border region from January 2017 to December 2018. We evaluated its performance on noise-level, dynamic range (DR), useful resolution (NU), collocated recording comparison, and shake map generation. The results proved that GL-P2B can be classified as a type of Class-B sensor. The records obtained are consistent with the data obtained by the collocated traditional force-balanced accelerometers even for stations with an epicenter distance of more than 150 km, and most of the relative percentage difference of peak ground acceleration (PGA) values is smaller than 10%. In addition, with the current density of the GL-P2B seismic network, near-real-time refined shake maps without using values derived for virtual stations could be directly generated, which will significantly improve the capability for earthquake emergency response. Overall, this MEMS-based sensor can meet the requirements of dense EEW purpose and lower the total investment of the National System for Fast Seismic Intensity Report and Earthquake Early Warning project.

## 1. Introduction

As one of the effective means for mitigating earthquake disaster, earthquake early warning (EEW) has been studied in many countries and regions all around the world [1,2,3,4,5,6,7,8,9]. Presently, few countries and regions already have operational EEW systems, such as Mexico, Japan, and Taiwan [10,11,12,13,14,15,16]. These systems have shown their great potentials in earthquake disaster reduction. In addition, in other parts of the world, some countries are developing and validating EEW systems, like southern Italy [17,18], California [19,20], China [21,22], Korea [23], and the Ibero-Maghrebian region [24].

Usually, because of the high cost of the installed broad-band seismometers or force-balanced accelerometers, the number of the sensors generally deployed in these EEW systems is relatively low. This would lead to poor azimuthal coverage and considerable location estimate errors. Without accurate epicenter location estimation, EEW systems cannot generate ground-motion shake with sufficient accuracy. They are then unable to issue timely alerts to the target areas. Although in a few of regions around the world like Taiwan and Japan, it has been proven that the station density of their seismic networks is rather enough for EEW and rapid reporting targets [25], we always demand a much higher density of seismic stations for EEW systems. However, for augmenting the number of stations deployed with conventional broad-band or force-balanced seismometers, this will cause to significantly increase the expense invested on EEW systems. For example, China is now building the National System for Fast Seismic Intensity Report and Earthquake Early Warning (NSFSIREEW) project. To cover the key seismic zones with an average interstation distance of about 10–12 km, more than 15,000 sensors are needed. If all the stations are deployed with high-cost traditional seismometers, the total investment of the project will be very high, approximately $1 billion. Therefore, a more acceptable way to carry out this project is to deploy a cost-effective seismic network.

A new type of sensors using low-cost Micro Electro Mechanical System (MEMS) has been used in seismic applications since the 1990s [26]. This sensor with a very small footprint can measure relative gravitational changes and provide an ideal, cost-saving solution to record high-frequency, near-field, and unsaturated strong ground shaking. Therefore, it offers a suitable application to creating an economical EEW system with high-density and large-scale seismic networks. Recently, several types of such sensors have been developed for seismological investigations, like the Quake-Catcher network [27], the SOSEWIN [28], the Palert network [29], the EDAS-MAS [30], and the MNSMS [31]. The gained results have presented their potentials for EEW.

However, according to the classification proposed by the Advanced National Seismic System (ANSS) [32], most of them with low dynamic range (DR) belong to the Class-C type. If we use these sensors in the EEW systems, they will introduce high biases in EEW parameters estimation, especially in estimating those frequency parameters, such as *τ_c_* and *τ_log_*, which need data with high signal-to-noise ratio (SNR). The detailed test results about some conventional and MEMS-based sensors can be found in Evans’ and Ringler’s study [33,34].

For improving the SNR of the recorded waveforms, we developed a new type of sensor called GL-P2B [35] with MEMS-integrated data logger and built-in seismological processing. It used a high DR MEMS which can reach 98 dB. The total price of mass production was lower than $600, approximately one tenth of a traditional strong-motion accelerometer [34]. After the system development completion, we deployed more than 10 stations for real field test. The obtained results demonstrated its feasibility in recording high-quality earthquake waveforms with *M*_L_ < 1.5 and a high SNR at distances beyond 50 km for earthquakes of *M*_L_ 3.0 or more. However, during the test period, we found that the CPU load was very high and it is impossible for us to add more functions into the system for meeting requirements of the NSFSIREEW project. Therefore, we upgraded the sensor with a new high-performance CPU board whose processing capacity was equivalent to twice that of the original one. In addition, some errors found during the test period were corrected. After the sensor upgrade was complete, a demonstration EEW project proposed by the China Earthquake Administration (CEA) was launched in 2015 for exploring the scientific EEW feasibility and providing experiences for the NSFSIREEW project. In this project, approximately 170 GL-P2B stations with an average interstation distance of 10 km were deployed in the Sichuan-Yunnan border region for more than two years.

In this research, with the collected noise data and recorded numerous earthquakes waveforms, we presented the concept and the results of this MEMS-based sensor network during the test period from January 2017 to December 2018. The performances were evaluated according to noise-level, dynamic range (DR), useful resolution (NU), collocated recording comparison, and shake map generation. The obtained results have proved that this MEMS-based sensor is sufficient for EEW purpose and can significantly lower the total investment of the NSFSIREEW project.

## 2. System Design and Implementation

**Figure 1:**
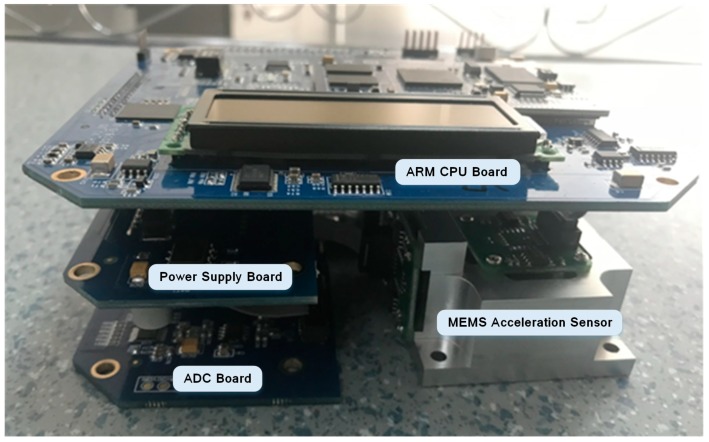
Hardware components of GL-P2B.

Here, we only presented a brief description of the hardware structure of the GL-P2B. Detailed information of the hardware and software functions description can be found in Peng’s research [35]. A GL-P2B is composed of four hardware parts: The MEMS acceleration sensor, an analog-to-digital converter (ADC) board, an ARM CPU board, and a power supply module. The composition of the device is shown in Figure 1.

**Figure 2:**
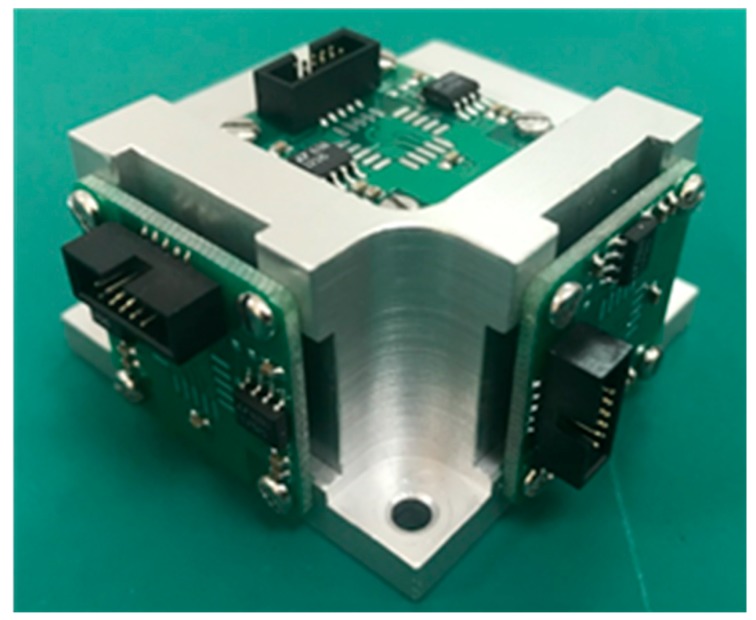
Photo of the Micro Electro Mechanical System (MEMS) acceleration sensor, in which three MSV6000-02 accelerometers are mounted orthogonally to each other.

To record seismic events in a three-dimensional space, the MEMS acceleration sensor is designed with three MSV6000-02 [36] capacitive (MT Microsystems Co., Ltd., Shijiazhuang, Hebei, China) MEMS accelerometers mounted orthogonally to each other (Figure 2). The main characteristics of the MSV6000-02 are: (1) Full scale acceleration range of ±2 g; (2) large and flat frequency response (± 5%) between 0–250 Hz; (3) noise density of 10 μV/Hz; (4) non-linearity <0.5% of full scale; (5) bias and sensitivity temperature coefficient of 0.2 mg/°C and 100 ppm/°C; (6) resolution/threshold (@1 Hz) of 0.002 mg.

Generally, low-cost MEMS sensors with a maximum resolution of 16 bits can be used to detect moderate to large seismic events within a distance of several tens of kilometers [33]. However, these types of sensors are not suitable for accurately picking *P*-wave of small earthquakes and estimating EEW parameters. Therefore, we selected a 24-bit ADC ADS1281 (Texas Instruments Incorporated, Dallas, Texas, USA) [37] for data acquisition and conversion.

For improving the CPU processing capacity, we upgraded the ARM CPU board and selected Atmel SAMA5D36 (Atmel Corporation, San Jose, CA, USA) as the core processor [38]. This CPU is a high-performance, power-efficient embedded MPU based on the ARM Cortex-A5 processor which can achieve 536 MHz with power consumption levels below 0.5 mW in low-power mode. The Linux 3.6.9 operating system is run on the CPU board. A 256 MB NAND Flash (Spansio, San Jose, CA, USA) is used for storing the YAFFS file system compiled within BusyBox 1.13.0, and a 8 GB+ embedded multimedia card (Emmc, Toshiba, Tokyo, Japan) is selected to act as a hard disk for waveform storage. The SAM-BA tools is adopted to erase the NAND flash and write the uboot, Linux kernel image, and UBI rootfs to the specified address of the NAND flash memory.

## 3. Performance Evaluation

### 3.1. Station Deployment

**Figure 3:**
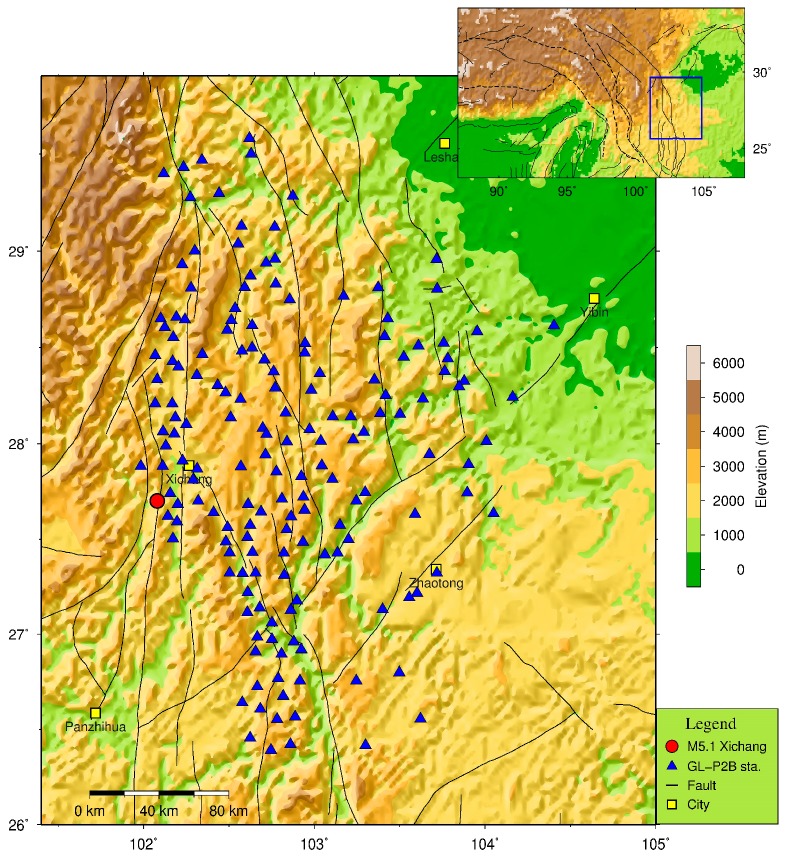
Station distribution of the GL-P2B based seismic sensor array shown as blue triangles. The red circle represents the epicenter of the 31 October 2018, *M* 5.1 Xichang earthquake. A large map with the marked study region is shown in the inset. Black lines indicate faults reported by Deng’s research [39].

With the support of the Department for Earthquake Monitoring and Prediction, CEA, a demonstration project with approximately 170 GL-P2B stations have been built and configured in the Sichuan-Yunnan border region since 2015. The station distribution of this project is shown in Figure 3. About 110 GL-P2B sensors were deployed with a conventional station installation mode and powered by 120 W solar panels and a 100 Ah backup battery. The other 60 GL-P2B stations were co-located with the traditional force-balanced strong-motion stations and used the same pier and power supply provided for those stations. Therefore, the traditional strong-motion accelerometers could be selected as references for performance comparison with the co-located GL-P2B sensors. Here, the traditional strong-motion stations generally deployed with BBAS-2 (also named RefTek RT-147-01/3) [34]. The low-latency data packetizing function of the GL-P2B [35] was used for data transferring at an interval of 0.5 s, and 100 samples per second (sps) was set as the real-time data outputting sampling rate. For each station, a 3G/4G router was used to transmit the ground-motion data recorded by the network to a processing server at the Sichuan Earthquake Administration. Additionally, in each 3G/4G router, the Virtual Private Network (VPN) was configured to provide each sensor a virtual fixed Internet Protocol (IP) address for ease management and maintenance.

### 3.2. Noise-Level Analysis

**Figure 4:**
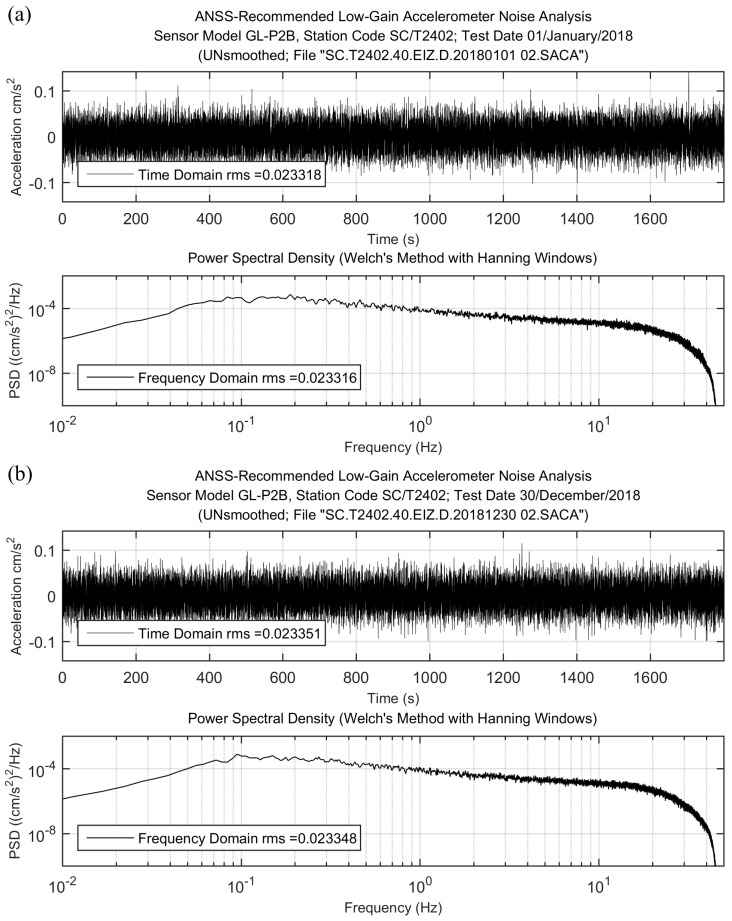
An example of noise data of the sensor SC/T2402 processed by a Butterworth bandpass filter between 0.1–20 Hz and the computed root mean square (RMS), power spectral density (PSD) values: (**a**) Results of the noise data obtained on 1 January 2018; (**b**) results of the noise data obtained on 30 December 2018.

In the seismology field, noise-level analysis is always considered as a power tool for evaluating the performance of a seismic device, from which the self-noise level, DR, and NU can be obtained. Here, we used the Matlab script “ANSS_noise_rms_rev4.m” recommended by ANSS to calculate root mean square (RMS) of the sensor self-noise and power spectral density (PSD). Two time periods (January 2018 and December 2018) were selected, and on these time periods we computed self-noise level for each GL-P2B sensor respectively. Following the suggestion of ANSS, we selected 30 min and 180,000 points for each channel at 100 sps in self-noise level computation. An example of results on 1 January 2018, and 30 December 2018, for the same GL-P2B sensor SC/T2402 is presented in Figure 4.

The recorded data shown in Figure 4 are the raw data obtained by the GL-P2B sensor SC/T2402 on 1 January 2018, and 30 December 2018, at 1 am and filtered by a Butterworth bandpass filter in the focused frequency band of 0.1–20 Hz. From Figure 4, we could find that there is no transient signals (such as earthquakes, instrumental glitches, or system artefacts) included. The offset of recorded data was removed, and most of the values are less than 0.5 cm/s^2^. From this result, we could confirm that the ambient noise is much less than the sensor noise-level, and the collected data are equal to the self-noise level of the GL-P2B sensor. In addition, RMS at time and frequency domains and PSD are computed. There is almost no difference between the values obtained in time and frequency domains. For each GL-P2B sensor, the RMS values were computed through three components: Up–down (UD), east–west (EW), and north–south (NS). Here, for comparison, we classified the RMS values of each sensor into three grades shown in Table 1. Table 2 shows RMS values of some GL-P2B stations as an example, including two stations with the grade-1 RMS values, two stations with the grade-2 RMS values, and two stations with the grade-3 RMS values.

For each sensor, RMS values of EW and NS components were almost the same, and the RMS value of UD was bigger than the other two components. This is in line with the structural characteristics of the MEMS accelerometer and can be found in all MEMS-based three axial sensors [33] because the UD component is influenced by a gravitational acceleration. In addition, the results obtained on 1 January 2018, and 30 December 2018, were similar to each other. It means that after 12 months of continuous work, the self-noise level of the GL-P2B sensors did not change, indicating that the performance of the GL-P2B was stable and reliable. 

After RMS values of all the GL-P2B stations were acquired, we calculated DR and NU of each sensor by using Equations (1) and (2):

(1) $$DR=20\mathrm{lg}\frac{0.707A}{RMS}$$

(2) $$NU=\frac{DR}{20\times \mathrm{lg}2}$$

where *A* is the clip level of the GL-P2B sensor, which is ±2 g. Here, according to the installation location of GL-P2B stations, we set the gravity acceleration as 9.7913 m/s^2^, which is the value obtained for the southwestern part of China [40]. The obtained results for each grade of GL-P2B stations are presented in Table 3.

As shown in Table 3, we could find that for the GL-P2B stations with the grade-1 and grade-2 RMS values, the differences in DR and NU were small. However, for those stations with the grade-3 RMS values, the maximum difference of RMS values, DR, and NU between the grade-2 and grade-3 was more than 0.01 cm/s^2^, 4 dB, and 0.8 bit, respectively. After carefully investigating those stations with grade-3 RMS values, we found that there were some noise sources near the stations. Some stations were too close to the highways, less than 100 m while other stations were too close to the processing factories. Therefore, for these stations, we need to move them to new sites with low ambient noise level for improving the data quality.

### 3.3. Collocated Recording Comparison

**Figure 5:**
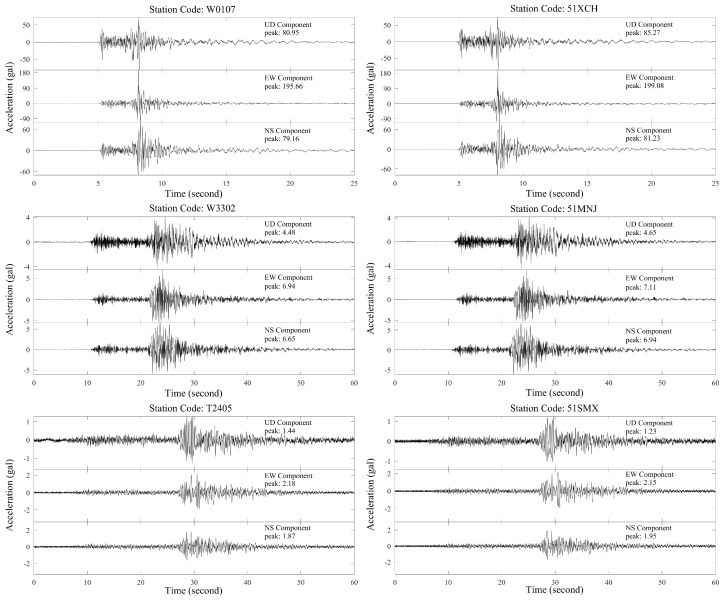
Examples of three-component accelerations from 31 October 2018, *M* 5.1 Xichang earthquake at the following co-located station pairs: W0107&51XCH, W3302&51MNJ, and T2405&51SMX. Stations 51XCH, 51MNJ, and 51SMX are traditional strong-motion stations with trigger-mode data transmission.

For those 60 GL-P2B sensors collocated with the traditional strong-motion stations, it provides an opportunity to check the data quality recorded by this low-cost seismic network. Each traditional strong-motion station uses a force-balanced accelerometer with a sampling rate of 200 Hz and a ±2 g full dynamic range. However, those strong-motion accelerometers are not upgraded and can only use the traditional trigger-mode for data transmission when the recorded ground-motion value of an earthquake reaches the predefined threshold (4 gal). It means that for the traditional trigger-mode stations, we cannot obtain the noise signals or waveforms for small earthquakes. We were lucky that during the test period, several earthquakes with *M* > 4.0 occurred in the study region. They provided us enough data for validating the data quality of the low-cost sensor. Here, in this research, we selected waveforms of the two largest earthquakes for comparison: 16 May 2018, *M* 4.3 Shimian, and 31 October 2018, *M* 5.1 Xichang earthquakes. Figure 5 shows the comparison of original waveforms recorded by three GL-P2B and traditional strong-motion station pairs for the Xichang earthquake: W0107&51XCH, W3302&51MNJ, and T2405&51SMX. The W0107&51XCH and T2405&51SMX station pairs have the nearest (16.6 km) and farthest (176.4 km) epicenter distances, respectively.

From Figure 5, we can find that the signals between these station pairs are almost the same as each other and all the features of the earthquake waveforms recorded on the three axial channels of the traditional accelerometers are perfectly duplicated by the proposed GL-P2B sensors, even for collocated stations with an epicenter distance of more than 150 km. Additionally, except the previous qualitative analysis, we also carried out quantitative assessments based on peak ground acceleration (PGA) estimate to further demonstrate the reliability of the proposed system. The capability of the proposed GL-P2B sensor to produce PGA or other strong-motion parameters is very important for civil protection and monitoring oriented engineering applications. Appendix A presents the results about the PGA parameter and its relative percentage difference of each collocated station for the two largest earthquakes with *M* 5.1 and *M* 4.3. The relative percentage difference of the PGA values is computed as follows:

(3) $$\delta PGA_{rel}=\frac{PGA_{tra}-PGA_{GL-P2B}}{PGA_{tra}}\times 100$$

where *PGA_tra_* and *PGA_GL-P2B_* are the values of the PGA parameter for the traditional strong-motion accelerometer and the GL-P2B sensor, respectively. The parameters referred to the collocated stations were computed for each of the three acceleration channels (UD, EW, and NS). The estimates of the PGA values are similar for *M* 4.3 and *M* 5.1 earthquakes, and most of the *δPGA_rel_* are smaller than 10%. The PGA values generally decrease as the magnitude of the earthquake and the epicenter distance of the station decrease, in line with the ground-motion attenuation characteristics. For those components with relative percentage difference higher than ±20%, we found that there were errors on the sensitivity calibration coefficients of the GL-P2B stations. After recalibrating the sensitivity coefficients, the relative percentage difference would be less than 10%.

### 3.4. Shake Map Generation

Shake map is considered as one of the powerful tools for earthquake emergency response purposes. The more detailed the shake map, the better the effect we can obtain. Generally, the shake map is produced with peak ground-motion parameters (PGA or others) from real and virtual stations. The latter is derived from ground-motion prediction equations (GMPE). When the seismic network in the earthquake source region is sparse, the values of the virtual stations will be the control factor, leading to large system biases compared with the real ones. Traditionally, more than ten minutes are needed for generating a shake map for an earthquake because we firstly need to collect the trigger-mode event waveforms and then locate the epicenter and estimate the magnitude. However, with real-time transmission of strong-motion signals recorded by the GL-P2B sensor network, we can generate a near-real-time shake map immediately after an earthquake being detected. This would significantly shorten the time needed when compared with that produced by the traditional trigger-mode strong-motion network.

**Figure 6:**
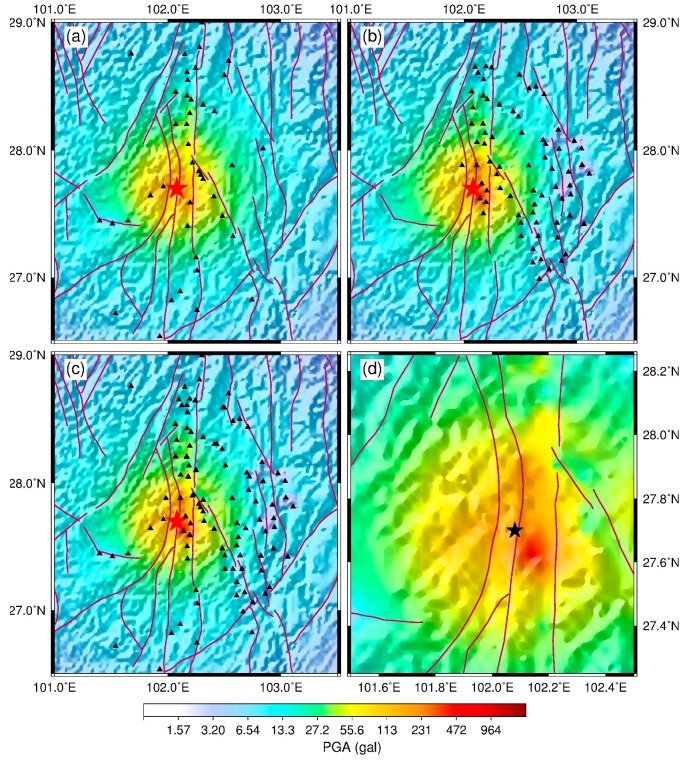
Peak ground acceleration (PGA) shake maps for the 31 October 2018, *M* 5.1 Xichang earthquake produced by (**a**) traditional trigger-mode strong-motion stations, (**b**) the GL-P2B sensor network, and (**c**) the combined networks (traditional trigger-mode strong-motion and GL-P2B stations). (**d**) PGA shake map for the earthquake source regions of the Xichang earthquake produced by the combined networks. The triangles represent the distribution of traditional and GL-P2B stations, while the star indicates the epicenter of this earthquake.

Figure 6 compares the PGA shake map using the GL-P2B network with the traditional one for the 31 October 2018, *M* 5.1 Xichang earthquake. Because this earthquake was located at the edge of the GL-P2B sensor network, the values derived for the virtual stations controlled the western part of the PGA shake map. However, relative to the result based on the traditional strong-motion stations (Figure 6a), the eastern part of the PGA shake map using the GL-P2B network was more detailed because of higher density of this network in this region. In the earthquake source region (orange-red areas in the middle), five GL-P2B stations observed a PGA higher than 100 gal with one showing a PGA approximately 400 gal, while only one traditional station recorded a PGA higher than 100 gal. Using the combined sensor network, we could obtain a more detailed shake map for this earthquake (Figure 6c,d), although the differences among Figure 6a–c are not apparent because the density of the traditional strong-motion stations in the source area was high.

**Figure 7:**
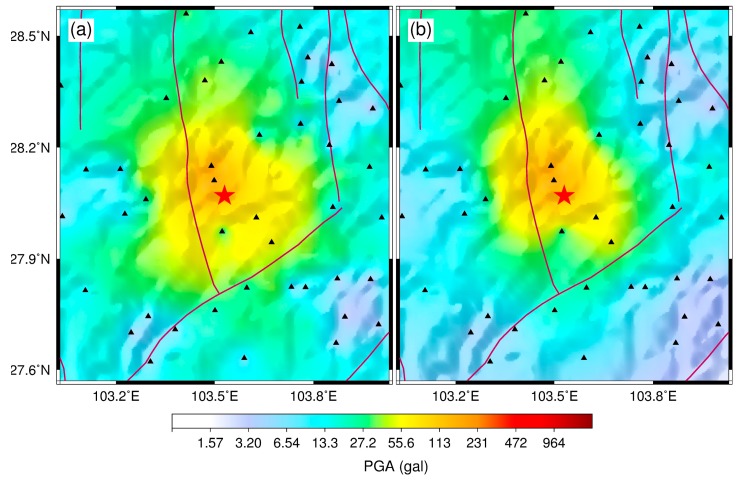
PGA shake maps for the 16 May 2018, *M* 4.3 Shimian earthquake (**a**) with and (**b**) without virtual stations. PGA values for the virtual stations were derived using the ground-motion prediction equation proposed in Yu’s study [41]. The triangles represent the GL-P2B stations, while the star indicates the epicenter of this earthquake.

The 16 May 2018, *M* 4.3 Shimian earthquake was selected as an example to check whether we could directly use the GL-P2B sensor network without virtual stations to generate a PGA shake map for an earthquake. The source region of this event was well surrounded by the low-cost seismic network. As shown in Figure 7, one can find that the result without using virtual stations is more reasonable because the values derived from the GMPE usually contain system biases. Therefore, a dense array deployed with low-cost MEMS sensors is very useful for this purpose.

**Figure 8:**
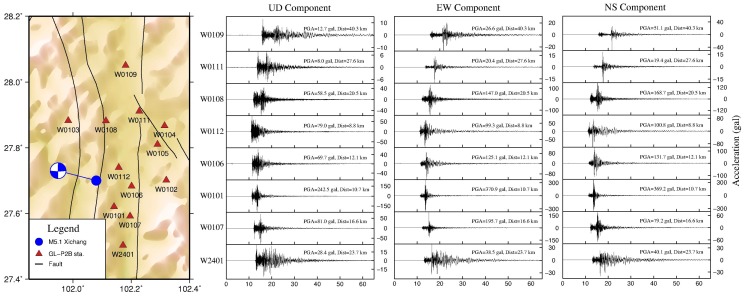
A series of acceleration waveforms recorded by GL-P2B sensor network during the 31 October 2018, *M* 5.1 Xichang earthquake. These plots show the data quality of the network for up–down (UD), east–west (EW), and north–south (NS) components.

The detailed shake map can as be used to identify rupture direction which is one of the key factors for estimating possible damage after an earthquake occurs. Currently, traditional methodologies require more analysis time to estimate the rupture direction. Figure 8 presents a series of acceleration waveforms from GL-P2B stations close to the *M* 5.1 Xichang earthquake. Relative to the EW direction, strong motion waveforms in the NS direction show relatively larger amplitudes except three PGA values in the EW direction. Therefore, ruptures could proceed from the hypocenter along two directions. One rupture was along the north and the other went south, leading to high seismic intensity distributed in the north–south direction. This is consistent with the long axis direction of the intensity map published by the Sichuan Earthquake Administration [42]. Thus, using a detailed shake map, we can potentially identify the earthquake rupture direction. This represents significant progress in earthquake observations for the test region.

## 4. Conclusions

For increasing the density of the seismic network for EEW purpose, a tri-axial sensor with high-dynamic MEMS named GL-P2B was developed. This sensor was an upgraded version from the previous one by improving the CPU processing capability and correcting some errors found during the initial test period. To fully reveal its performance after upgrading, approximately 170 GL-P2B sensors were installed and tested in the Sichuan-Yunnan border region from January 2017 to December 2018. The field test site is located in an area with a high seismic risk, and several earthquakes with *M* > 4.0 were recorded. In this research, we evaluated the sensor’s performances from noise-level, DR, NU, collocated recording comparison, and shake map generation.

With the collected data, we obtained the ANSS-recommended self-noise of the GL-P2B sensor. For the stations with grade-1 and grade-2 RMS values, the differences in DR and NU were small, less than 2 dB and 0.5 bit, respectively. Some stations with grade-3 RMS values were influenced by some noise sources near them, suggesting site movement for data quality improvement. After two years of continuous work, the noise-level changing rates of most GL-P2B sensors are less than 10%, indicating that this sensor’s performance is stable and reliable. Based on the results of DR and NU, we confirmed that GL-P2B can be classified as a type of Class-B sensor.

The earthquake signals recorded by GL-P2B and collocated traditional strong-motion accelerometers are almost the same as each other and all the features of the earthquake waveforms recorded on the three axial channels of the traditional ones are perfectly duplicated by the proposed GL-P2B sensors, even for collocated stations with epicenter distance more than 150 km. And most of the relative percentage difference of the PGA values are smaller than 10%, suggesting that the records obtained by the GL-P2B sensors are consistent with the data obtained by the traditional strong-motion accelerometers. Those stations with relative percentage difference higher than 20% were caused by errors on the sensitivity calibration coefficients, which will be solved by recalibrating them.

Relative to the traditional trigger-mode strong-motion stations, with the current density of the GL-P2B seismic network, we could directly draw near-real-time refined shake maps for earthquakes occurred in this network without using values derived for virtual stations. In addition, based on the refined shake map, the rupture direction for an earthquake can be potentially identified. This will significantly improve the capability for disaster risk reduction, earthquake emergency preparations and response.

In conclusion, the real field test results proved that this MEMS-based sensor can meet the requirements of dense EEW purpose and can significantly lower the total investment of the NSFSIREEW project from $1 billion to $0.3 billion. Moreover, in terms of its high data quality, it can also be used in other seismological applications, like structural health monitoring.

## Figures and Tables

**Table 1:** Definition of RMS value classification for each GL-P2B station.

Grade	UD RMS Values (cm/s^2^)	EW RMS Values (cm/s^2^)	NS RMS Values (cm/s^2^)
grade-1	<0.023	<0.019	<0.019
grade-2	0.023–0.030	0.019–0.026	0.019–0.026
grade-3	>0.030	>0.026	>0.026

**Table 2:** RMS values for some GL-P2B stations.

No.	Grade	Station Code	Time: 1 January 2018, at 1 am RMS Values (cm/s^2^)			Time: 30 December 2018, at 1 am RMS Values (cm/s^2^)		
			UD	EW	NS	UD	EW	NS
1	grade-1	SC/W0113	0.0193	0.0165	0.0179	0.0192	0.0167	0.0180
2	grade-1	YN/C2515	0.0210	0.0157	0.0176	0.0211	0.0157	0.0177
3	grade-2	YN/C2105	0.0261	0.0194	0.0200	0.0271	0.0219	0.0211
4	grade-2	SC/W3702	0.0261	0.0199	0.0200	0.0255	0.0201	0.0198
5	grade-3	SC/W3701	0.0366	0.0277	0.0259	0.0330	0.0259	0.0258
6	grade-3	SC/W0105	0.0361	0.0296	0.0278	0.0388	0.0307	0.0293

**Table 3:** Estimated dynamic range (DR) and useful resolution (NU) of each grade of the GL-P2B stations.

Grade	Component	RMS Values (cm/s^2^)	DR/dB	NU/bits
grade-1	UD	0.0193–0.0229	95.63–97.12	15.9–16.1
	EW	0.0157–0.0189	97.30–98.91	16.2–16.4
	NS	0.0160–0.0188	97.34–98.74	16.2–16.4
grade-2	UD	0.0230–0.0300	93.28–95.59	15.5–15.9
	EW	0.0190–0.0260	94.53–97.25	15.7–16.2
	NS	0.0191–0.0259	94.56–97.21	15.7–16.1
grade-3	UD	0.0303–0.0388	91.05–93.20	15.1–15.5
	EW	0.0262–0.0297	93.37–94.46	15.5–15.7
	NS	0.0261–0.0279	93.92–94.49	15.6–15.7

## Appendix A

Table A1 presents the results about the PGA parameter and its relative percentage difference of each collocated station for the two largest earthquakes with *M* 5.1 and *M* 4.3.

**Table A1:** Relative differences of peak ground acceleration (PGA) recorded by collocated GL-P2B and traditional strong-motion stations for the two largest earthquakes with *M* 5.1 and *M* 4.3.

*M*	Epicenter Distance (km)	Strong-motion Station Code	Comp.	PGA (cm/s^2^)	GL-P2B Station Code	PGA (cm/s^2^)	δPGA_rel_ (%)
5.1	16.6	51XCH	UD	85.27	W0107	80.95	5.07
			EW	199.08		195.66	1.72
			NS	81.23		79.16	2.55
	24.0	51XCN	UD	31.04	W0105	31.01	0.10
			EW	40.68		40.60	0.20
			NS	56.80		53.30	6.16
	27.6	51XCX	UD	8.02	W0111	8.00	0.27
			EW	18.90		20.44	−8.11
			NS	18.84		19.44	−3.15
	33.2	51PGW	UD	9.53	W2810	9.45	0.83
			EW	20.21		20.86	−3.25
			NS	25.66		25.94	−1.10
	40.3	51XCL	UD	13.39	W0109	12.73	4.96
			EW	27.73		26.64	3.94
			NS	50.07		51.09	−2.04
	46.1	51PGQ	UD	8.70	W2809	7.32	15.85
			EW	32.55		31.62	2.86
			NS	27.05		27.02	0.09
	56.5	51MNZ	UD	8.04	W3310	10.06	−25.04
			EW	28.07		29.12	−3.74
			NS	18.33		17.85	2.62
	57.1	51MNM	UD	16.95	W3307	16.11	4.97
			EW	37.25		36.90	0.94
			NS	38.76		38.71	0.13
	75.6	51XDG	UD	1.20	W3203	1.77	−47.87
			EW	5.38		5.33	1.08
			NS	3.15		3.24	−2.92
	76.5	51XDM	UD	4.75	W3207	5.45	−14.80
			EW	11.94		11.38	4.67
			NS	9.78		9.79	−0.07
	78.7	51MNS	UD	5.09	W3305	4.94	3.05
			EW	19.60		19.49	0.53
			NS	11.12		11.35	−2.07
	81.5	51MNF	UD	7.23	W3303	7.25	−0.26
			EW	13.48		14.00	−3.88
			NS	12.31		12.09	1.76
	84.6	51MNH	UD	10.14	W3306	9.75	3.88
			EW	15.80		15.29	3.21
			NS	13.00		12.54	3.53
	95.4	51MNJ	UD	4.65	W3302	4.48	3.66
			EW	7.11		6.94	2.39
			NS	6.94		6.65	4.18
	105.7	51MNA	UD	5.03	W3311	5.15	−2.39
			EW	14.62		14.83	−1.40
			NS	12.68		12.96	−2.22
	106.3	51MNC	UD	3.09	W3301	3.16	−2.26
			EW	7.57		7.52	0.71
			NS	7.92		7.76	1.98
	106.8	51YXZ	UD	1.85	W3408	1.83	0.95
			EW	8.11		8.01	1.26
			NS	5.50		6.61	−20.22
	107.0	51MND	UD	1.72	W3312	1.81	−5.15
			EW	5.04		4.46	11.47
			NS	5.63		5.54	1.50
	120.1	51YXX	UD	2.17	W3406	2.33	−7.08
			EW	3.37		3.43	−1.95
			NS	4.58		4.89	−6.70
	124.8	51MNW	UD	2.80	W3308	2.10	25.21
			EW	3.69		3.73	−1.01
			NS	4.88		4.91	−0.61
	146.3	51SML	UD	2.39	T2403	2.48	−3.86
			EW	7.33		7.21	1.63
			NS	7.80		7.48	4.19
	161.3	51SMC	UD	1.39	T2401	1.59	−14.39
			EW	3.47		3.31	4.61
			NS	3.49		3.30	5.44
	176.4	51SMX	UD	1.23	T2405	1.44	−16.82
			EW	2.15		2.18	−1.40
			NS	1.95		1.87	4.26
4.3	8.7	51SMC	UD	52.49	T2401	62.01	−18.14
			EW	165.16		157.71	4.51
			NS	197.69		189.23	4.28
	10.8	51SMX	UD	45.22	T2405	38.32	15.26
			EW	83.60		80.17	4.10
			NS	70.06		72.57	−3.57
	28.5	51SMK	UD	4.91	T2402	4.51	8.09
			EW	8.43		8.09	4.07
			NS	4.91		4.80	2.17
	41.3	51MNW	UD	6.89	W3308	8.32	−20.86
			EW	11.35		11.93	−5.08
			NS	16.27		16.03	1.51
	49.9	51HYJ	UD	5.84	T2301	5.76	1.39
			EW	5.10		4.63	9.32
			NS	5.18		4.89	5.48
	59.0	51YXX	UD	9.28	W3406	9.96	−7.34
			EW	21.07		21.45	−1.83
			NS	35.28		33.82	4.13
	59.5	51MNC	UD	4.13	W3301	4.80	−16.16
			EW	6.45		6.41	0.66
			NS	6.39		6.18	3.26
	61.2	51MNA	UD	3.42	W3311	3.40	0.60
			EW	5.34		5.94	−11.20
			NS	6.70		6.98	−4.30
	87.1	51MNS	UD	2.12	W3305	2.17	−2.33
			EW	3.32		3.37	−1.74
			NS	2.75		2.81	−2.12
